# Supplementary material for: An Integrative Study Showing the Adaptation to Sub-Optimal Growth Conditions of Natural Populations of Arabidopsis thaliana: A Focus on Cell Wall Changes
Source: Cells. 2020 Oct 7;9(10):2249. doi: 10.3390/cells9102249 (PMC7601860; doi:10.3390/cells9102249)

Supplementary Figures

An integrative study showing the adaptation to sub-optimal growth conditions of natural populations of *Arabidopsis thaliana*: a focus on cell wall changes

Duruflé *et al.*

Figure S1. Phenotype of floral stems in contrasted growth conditions.

Figure S2. Phenotype of rosettes in contrasted growth conditions.

Figure S3. Reconstruction of the main cell wall polysaccharides of rosettes cell walls from monosaccharide analysis.

Figure S4. Reconstruction of the main cell wall polysaccharides of floral stems cell walls from monosaccharide analysis.

Figure S5. Individual plots project the rosette samples for the four blocks used for the sparse MB-PLS-DA analyses.

Figure S6. Graphical representation of sparse MB-PLS-DA analyses that discriminate the floral stems Col, Roch, Grip, Hern and Hosp)according to the growth temperature.

Figure S7 Graphical representation of sparse MB-PLS-DA analyses that discriminate the floral stem samples according to Col, Roch, Grip, Hern and Hosp.

Figure S1. Phenotype of floral stems in contrasted growth conditions. A, Mass; B, Diameter; C, Length.

Measurements were done before the harvest. For each condition, 20 plants from 3 independent batches were analyzed. Mean values are represented.

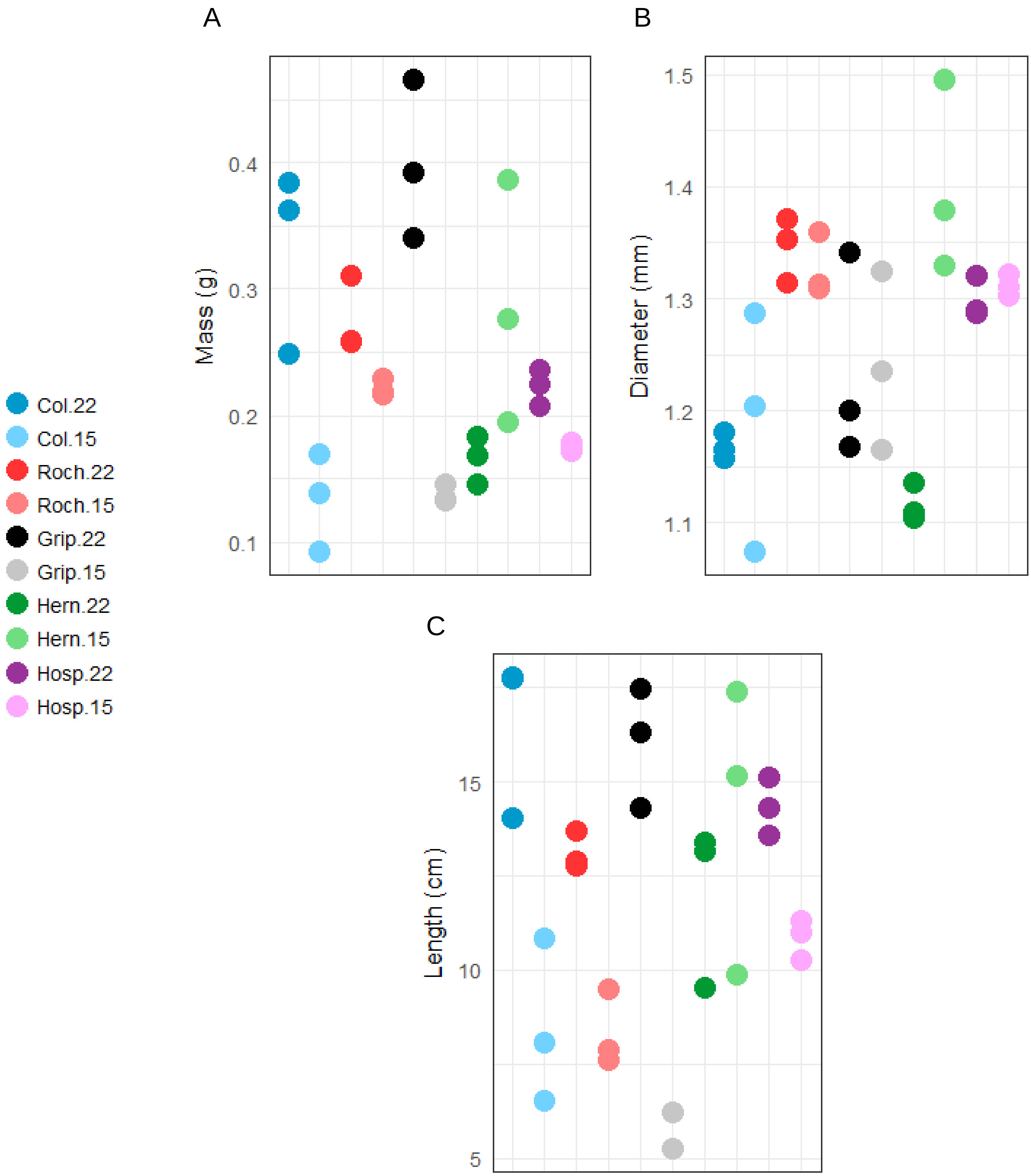

Figure S2. Phenotype of rosettes in contrasted growth conditions. A, Rosette diameter; B, Rosette mass; C, Rosette density; D, Projected rosette area.

Measurements were done at the time of harvesting. For each condition, 20 plants from 3 independent batches were analyzed. Mean values are represented.

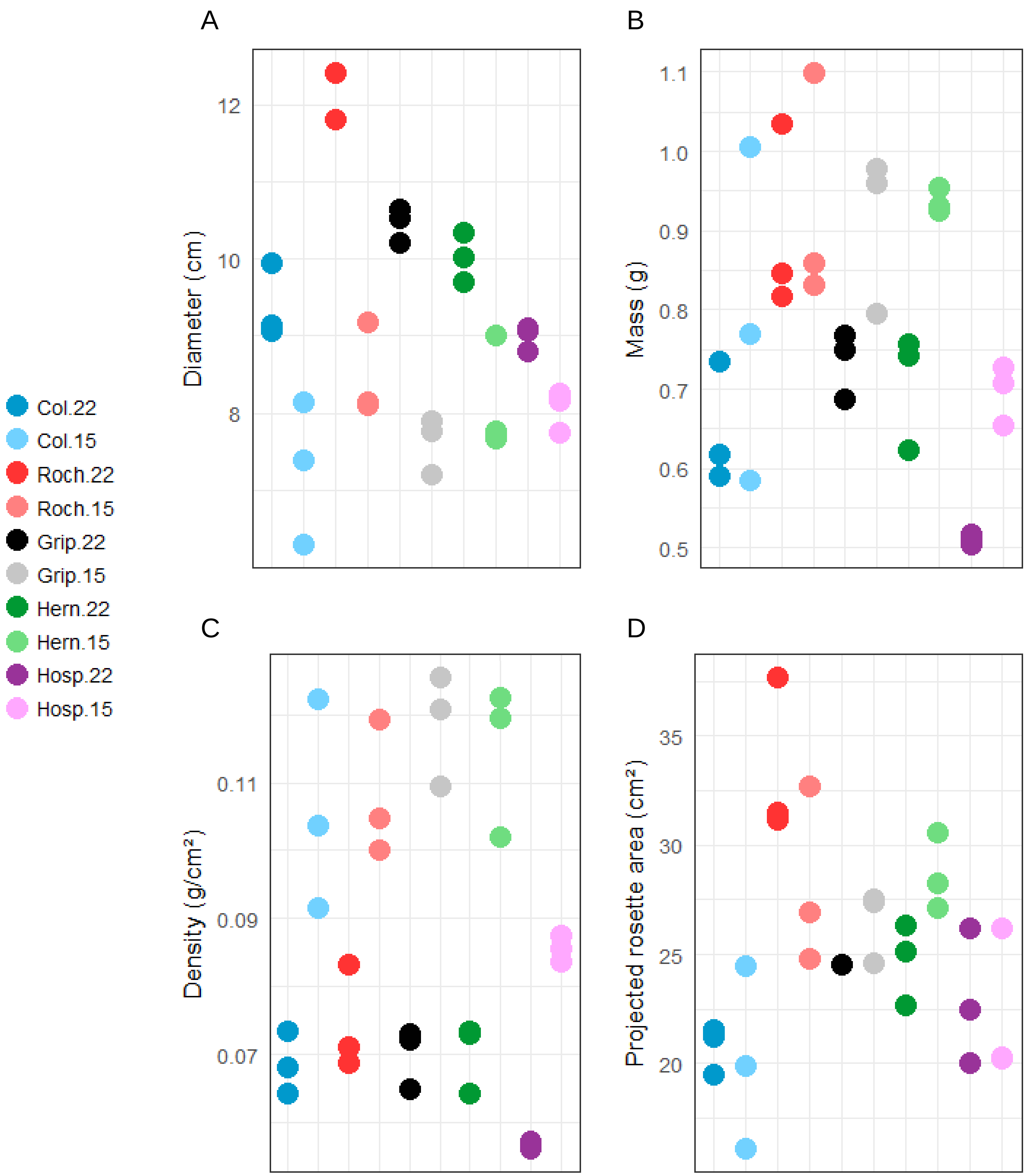

Figure S3. Reconstruction of the main cell wall polysaccharides of rosettes cell walls from monosaccharide analysis. Monosaccharide composition of rosettes of plants grown at 22 °C or 15 °C has been obtained. The formulas used to rebuild the polysaccharides are given in Supplementary Table S1. Mean values were calculated from 3 independent batches. XG: xyloglucans; RGI: rhamnogalacturonan I; HG: homogalacturonan.

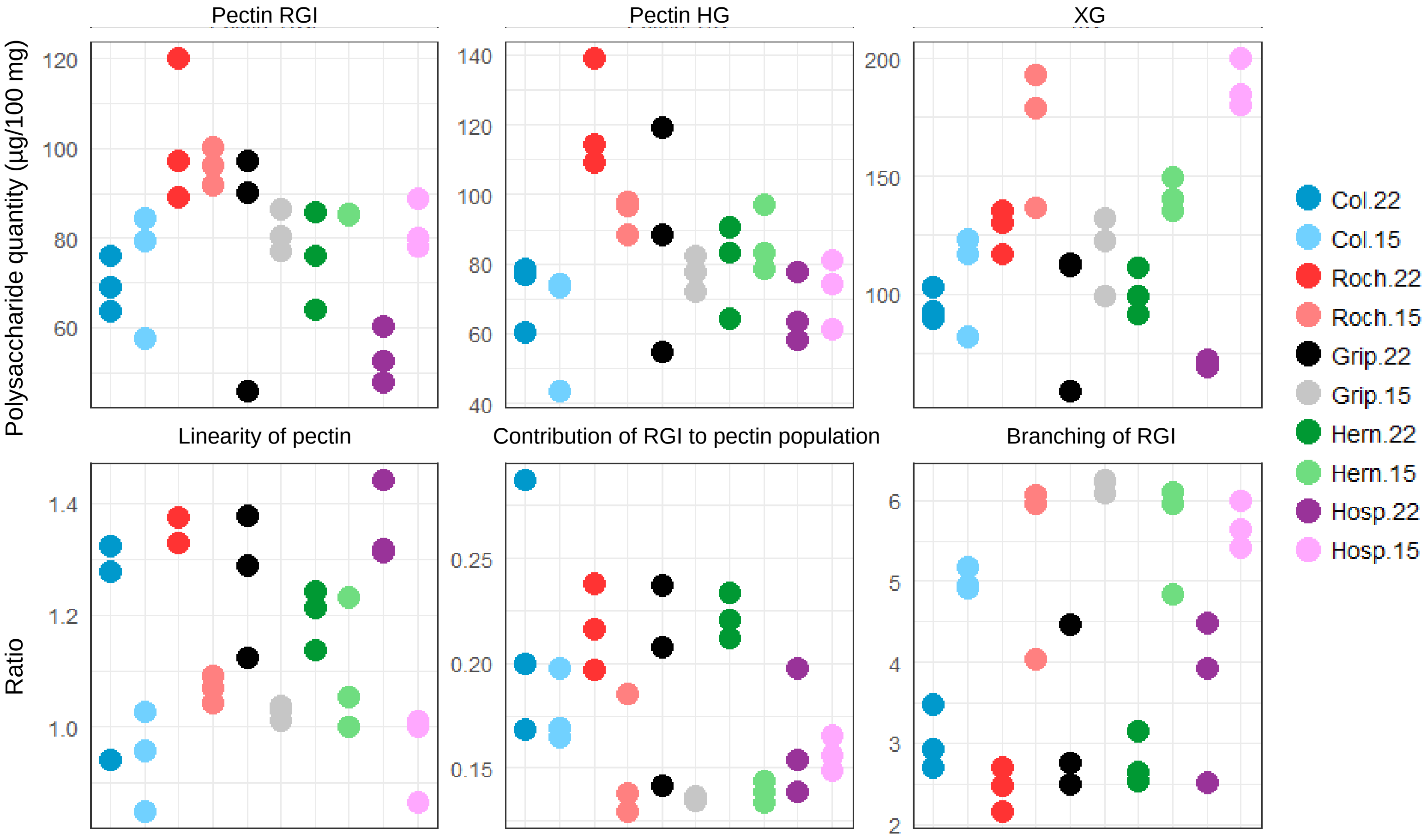

Figure S4. Reconstruction of the main cell wall polysaccharides of floral stems from monosaccharide analysis. Monosaccharide composition of floral stems of plants grown at 22 °C or 15 °C were obtained. The formulas used to rebuild the polysaccharides are given in Table S1. Mean values are calculated from 3 independent batches. XG: xyloglucans; RGI: rhamnogalacturonan I; HG: homogalacturonan.

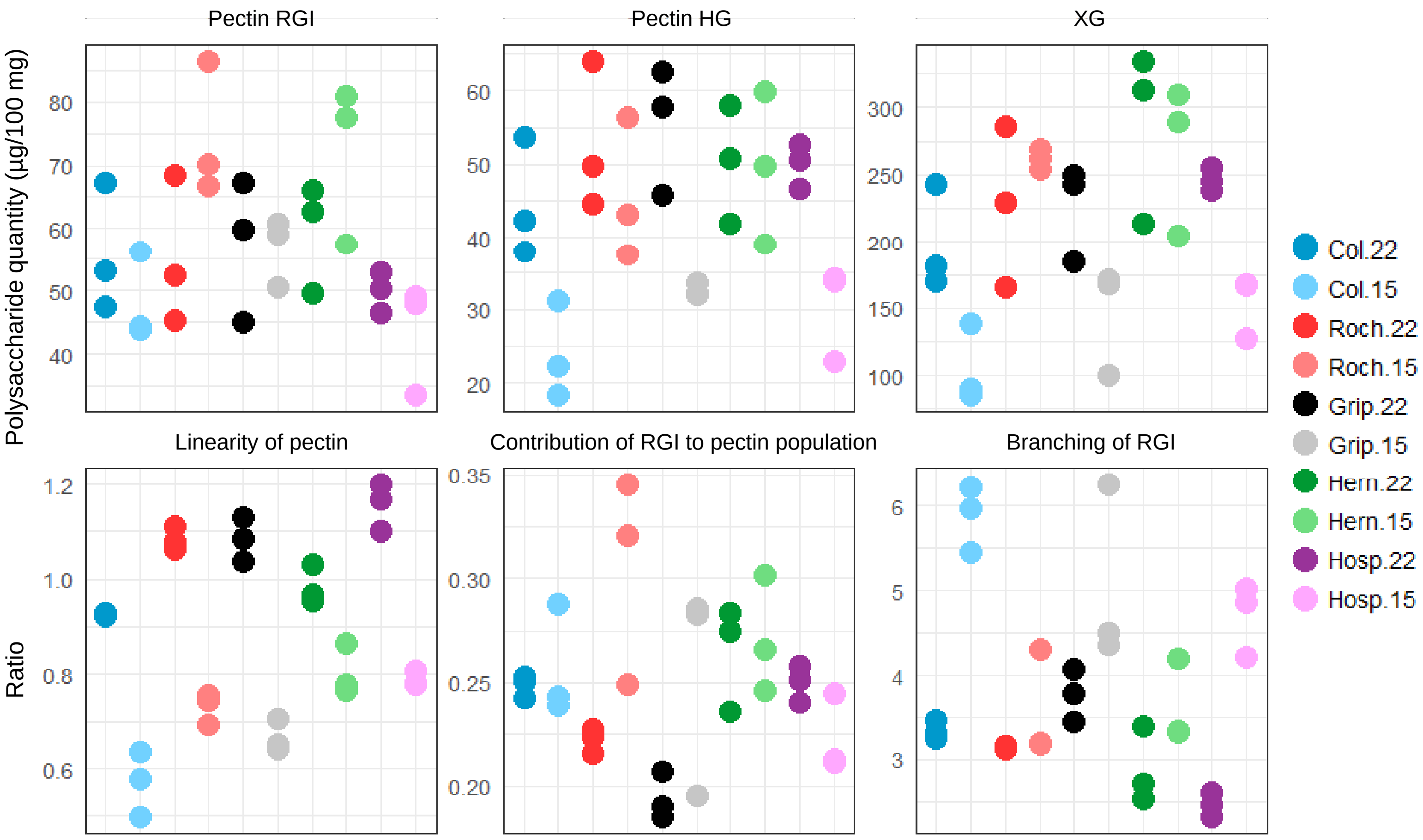

Figure S5. Individual plots project the rosette samples for the four blocks used for the sparse MB-PLS-DA analyses. They are discriminated according to (**A**) the growth temperature and (**B**) the five *A. thaliana* populations (Col, Roch, Grip, Hern and Hosp). Grey lines separate the samples according to the growth temperature (A).

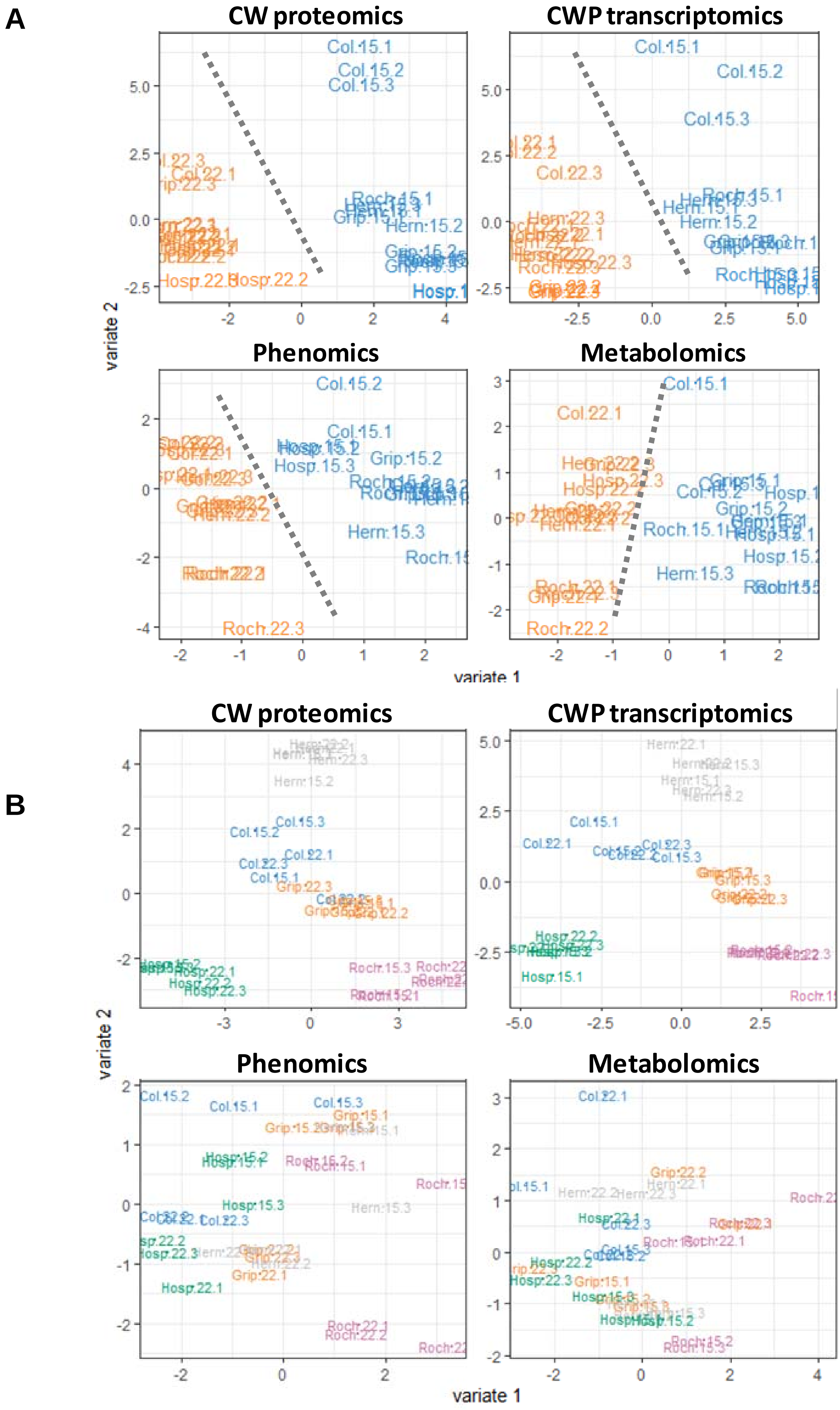

Figure S6. Graphical representation of sparse MB-PLS-DA analyses that discriminate the floral stems of the five *A. thaliana* populations (Col, Roch, Grip, Hern and Hosp) according to the growth temperature. (A) The plotDIABLO shows the correlation inside each block pair. (B) Individual plots projects the samples for the four blocks. Grey lines separate the samples according to the growth temperature.(C) Clustered image map representing the multi-omics profiles for each sample discriminated by the temperature: the levels of yellow and purple denote low value and high value respectively. Note that colors are scaled per line.

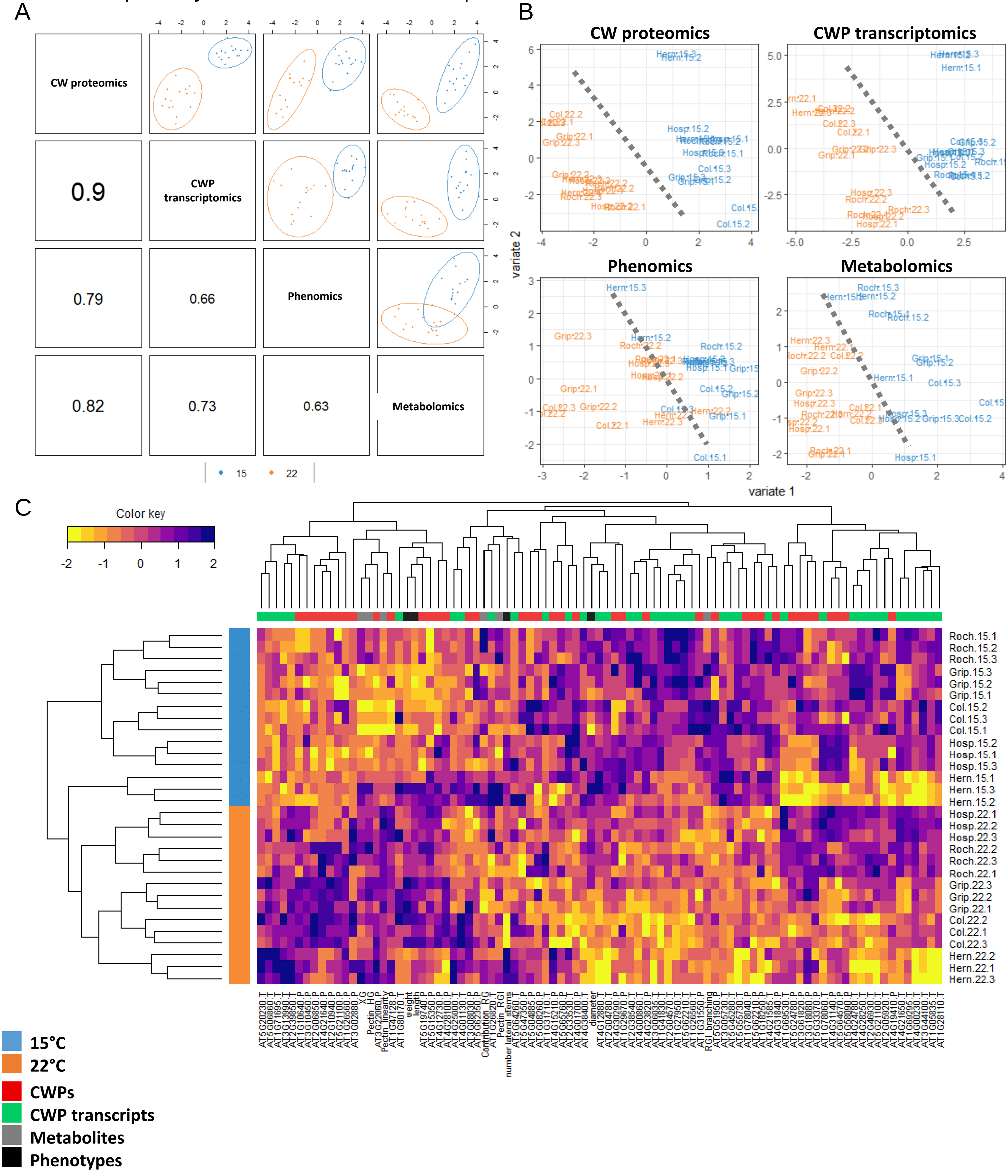

Figure S7 Graphical representation of sparse MB-PLS-DA analyses that discriminate the floral stem samples according to the five *A. thaliana* populations (Col, Roch, Grip, Hern and Hosp). (A) The plotDIABLO shows the correlation inside each block pair. (B) Individual plots project the samples for the four blocks. Colored ellipses highlight the Hosp and Hern samples. (C) Clustered image map representing the multi-omics profiles for each sample discriminated according to ecotypes: the levels of yellow and purple denote low value and high value respectively. Note that colors are scaled per line.

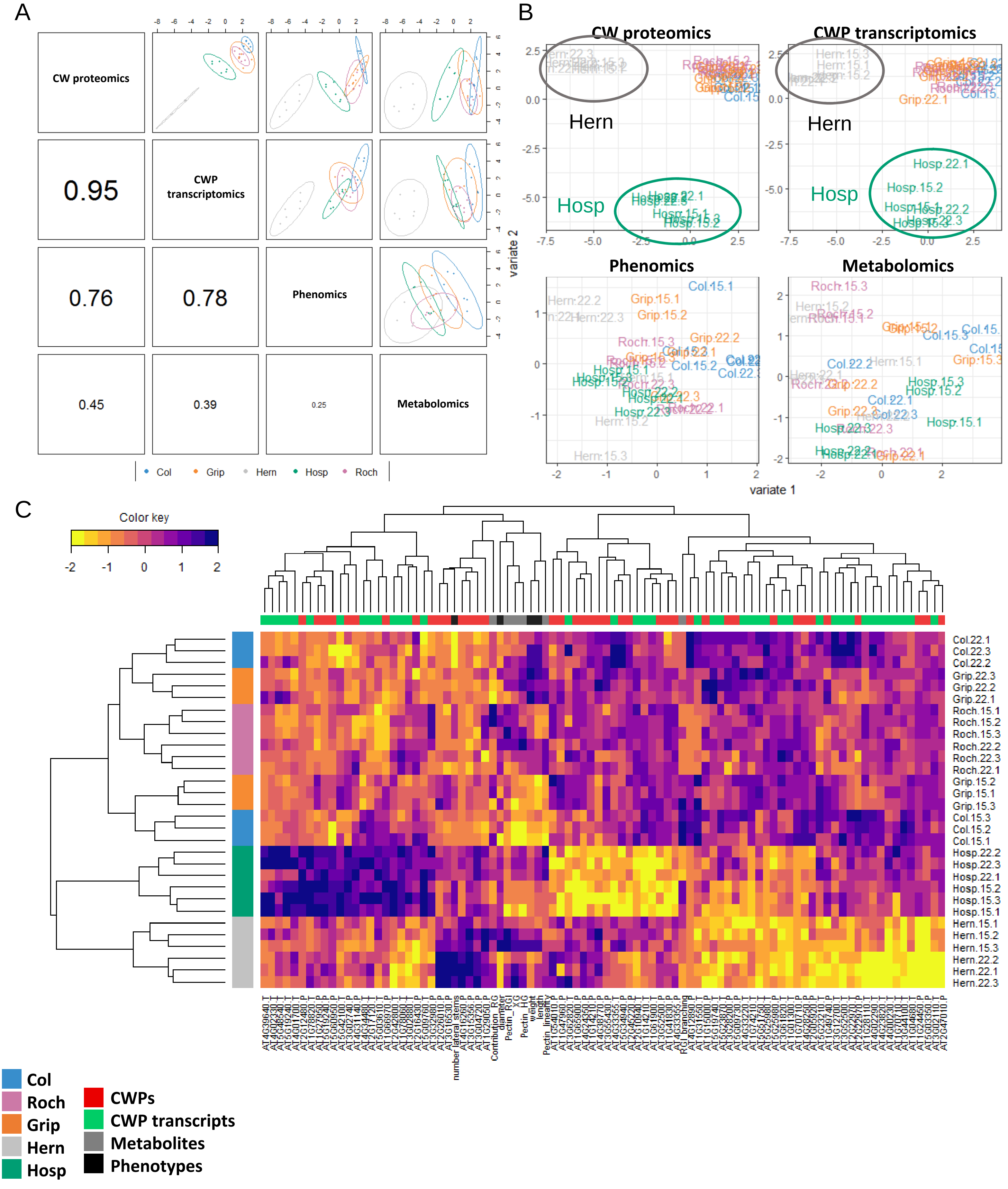

Supplement: Supplementary file 1 [file cells-09-02249-s001.zip › Duruflé et al._Supplementary Figures_R1.pdf]
